# Deep learning in prediction of intrinsic disorder in proteins

**DOI:** 10.1016/j.csbj.2022.03.003

**Published:** 2022-03-08

**Authors:** Bi Zhao, Lukasz Kurgan

**Affiliations:** Department of Computer Science, Virginia Commonwealth University, Richmond, VA 23284, USA

**Keywords:** Intrinsic disorder, Disordered regions, Disordered binding regions, Prediction, Deep learning, Deep neural networks, IDP, Intrinsically disordered protein, IDR, Intrinsically disordered region, DNN, Deep neural network, CASP, Critical Assessment of Structure Prediction, CAID, Critical Assessment of Intrinsic Protein Disorder, FFNN, Feed forward neural networks, BRNN, Bidirectional recurrent neural networks, CNN, Convolutional neural networks

## Abstract

Intrinsic disorder prediction is an active area that has developed over 100 predictors. We identify and investigate a recent trend towards the development of deep neural network (DNN)-based methods. The first DNN-based method was released in 2013 and since 2019 deep learners account for majority of the new disorder predictors. We find that the 13 currently available DNN-based predictors are diverse in their topologies, sizes of their networks and the inputs that they utilize. We empirically show that the deep learners are statistically more accurate than other types of disorder predictors using the blind test dataset from the recent community assessment of intrinsic disorder predictions (CAID). We also identify several well-rounded DNN-based predictors that are accurate, fast and/or conveniently available. The popularity, favorable predictive performance and architectural flexibility suggest that deep networks are likely to fuel the development of future disordered predictors. Novel hybrid designs of deep networks could be used to adequately accommodate for diversity of types and flavors of intrinsic disorder. We also discuss scarcity of the DNN-based methods for the prediction of disordered binding regions and the need to develop more accurate methods for this prediction.

## Graphical abstract

**Figure:**
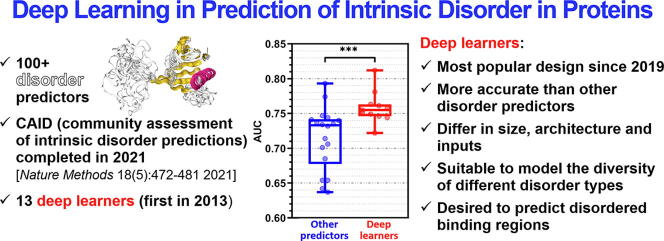

## 1 Introduction

Intrinsic disorder in proteins is defined by lack of stable tertiary structure under physiological conditions [1], [2], [3], [4]. Intrinsically disordered proteins (IDPs) include one or more intrinsically disordered regions (IDRs) in their sequences. Recent bioinformatics investigations conclude that IDPs are highly abundant in eukaryotic organisms [5], [6], [7] and enriched in multiple cellular compartments [8], [9]. Numerous studies of IDPs reveal that they are crucial for a wide spectrum of cellular functions that include signaling, molecular recognition and assembly, cell cycle regulation, transcription, translation and phase separation [10], [11], [12], [13], [14], [15], [16], [17], [18], [19]. Moreover, given their functional importance and prevalence in the human diseasome [12], [20], [21], [22], they serve as promising and currently underutilized leads for rational drug design efforts [23], [24], [25], [26], [27].

Experimentally characterized IDPs and IDRs can be collected from several databases, such as DisProt [28], PDB [29], IDEAL [30], DIBS [31], and MFIB [32]. However, these resources cover only a small fraction of IDPs, with the largest DisProt and PDB databases currently including about 2 thousand and 25 thousand IDPs, respectively [28], [33]. Compared to over 225 million protein sequences that are available in the newest 2021_04 release of UniProt [34], we have a long way to go to comprehensively identify and annotate IDPs and IDRs. Computational methods that accurately predict intrinsic disorder can be used to facilitate efforts to close this huge and growing knowledge gap. Computational predictors already made large impact on the intrinsic disorder field, by powering a rapid acceleration in the research on IDPs and IDRs [35]. They are also used across many areas including rational drug design [23], [24], [25], [26], structural genomics [36], [37], [38], and medicine [39], [40].

Development of computational predictors of disorder is a long-standing research problem. A recent survey has identified 103 disorder predictors that were developed over the last four decades [41]. Current surveys point to the long history of the disorder prediction area, providing invaluable insights concerning architectures of these methods, their availability, trends in their development efforts and approaches to comparatively evaluate their predictive performance [40], [41], [42], [43], [44], [45], [46], [47], [48]. Moreover, users and developers benefit from empirical studies that comparatively assess predictive quality of disorder predictors [33], [49], [50], [51], [52], [53], [54], [55], [56], [57], [58], [59]. These comparative studies include several community assessments, such as Critical Assessment of Structure Prediction (CASP) between CASP5 to CASP10 [53], [54], [55], [56], [57], [58] and Critical Assessment of Intrinsic Protein Disorder (CAID) [52]. The community assessments involve evaluation of predictors on blind test datasets (i.e., datasets that were not available to the authors of the predictors) by independent assessors who do not take part in the competitions utilizing tests and metrics that are widely accepted by the community.

The predictive architectures used to develop disorder predictors are typically divided into three categories [42], [43], [46], [47]: (1) sequence scoring functions; (2) machine learning models; and (3) *meta*-predictors. The first category uses additive and/or weighted functions, some of which are grounded in physical principles governing protein folding, to process the input protein sequence and sequence-derived structural and evolutionary information. Representative disorder predictors that fall into this category include FoldIndex [60], IUPred [61], [62], and IUPred3 [63]. The machine learning predictors apply models that are trained from data using a variety of machine learning algorithms, such as support vector machines [64], [65], [66], regression [67], conditional random fields [68], [69], [70], radial basis function networks [71], and shallow neural networks [36], [72], [73], [74], [75], [76]. Example popular machine learning predictors include DisEMBL [36], DISOPRED [75], [76], PONDR [73], and PrDOS [64]. The *meta*-predictors use multiple disorder predictions as inputs to re-predict disorder. The underlying rationale was to exploit potential complementarity among the input disorder predictions to generate a new prediction that would improve over the inputs. These efforts were also fueled by the availability of diverse sequence-scoring and machine learning predictors and studies that empirically show that well-designed meta predictors indeed produce predictions that outperform their inputs [77], [78], [79]. Representative example *meta*-predictors of disorder include metaPrDOS [80], MFDp [65], [81], [82], Cspritz [83], disCoP [77], [84], and MobiDB-lite [78]. We observe that some *meta*-predictors use machine learning algorithms (e.g., metaPrDOS [80] and MFDp [65]), which means that they can be cross-listed in both categories.

Results of CASP10, the most recent CASP community assessment that covers disorder prediction (i.e., subsequent CASP experiments do not include disorder predictions), reveal that the top three predictors belong to the machine learning (PrDOS and DISOPRED) and *meta*-predictor (MFDp) categories [58]. However, a recent survey notes a rapid influx of a new subfamily of machine learning methods that relies on deep neural networks (DNNs) after the first DNN-based method was released in 2013 [41]. DNNs differ from shallow neural networks, which were commonly used to implement disorder predictors in early 2000 s [36], [72], [73], [74], [75], [76], by use of multiple hidden layers and more sophisticated types of neurons and connections. The shift to the deep network models is motivated by their favorable levels of predictive performance when compared with the other types of disorder predictors. In particular, we observe that the best performing methods from the just completed CAID experiment [85], which include flDPnn [86], SPOT-Disorder2 [87], RawMSA [88] and AUCpred [89], rely on DNNs. Motivated by their growing numbers and success, we provide the first review of the DNN-based disorder predictors. We identify and summarize 13 DNN-based disorder predictors that were developed since 2013. We analyze trends in the development of these predictors and empirically compare predictive quality produced by the deep learners against the other types of disorder predictors based on results produced on blind test dataset from the CAID experiment. We also comment on future prospects in the development of the DNN-based disorder predictors.

## 2 Prediction of intrinsic disorder using deep learning

Nowadays, deep learning is widely used to develop methods that predict protein structure and function. Perhaps the most obvious example is protein structure prediction where deep learning models, such as AlphaFold, have deservedly dominated over other types of methods [90], [91], [92], [93]. Moreover, deep learning is utilized to predict other structural aspects of proteins, such as contacts [94], secondary structure [95] and torsional angles [96]. DNNs are also successfully applied to predict protein function [97], [98], [99], protein-drug interactions [100], [101], and functional sites [102], [103], [104].

**Fig. 1:**
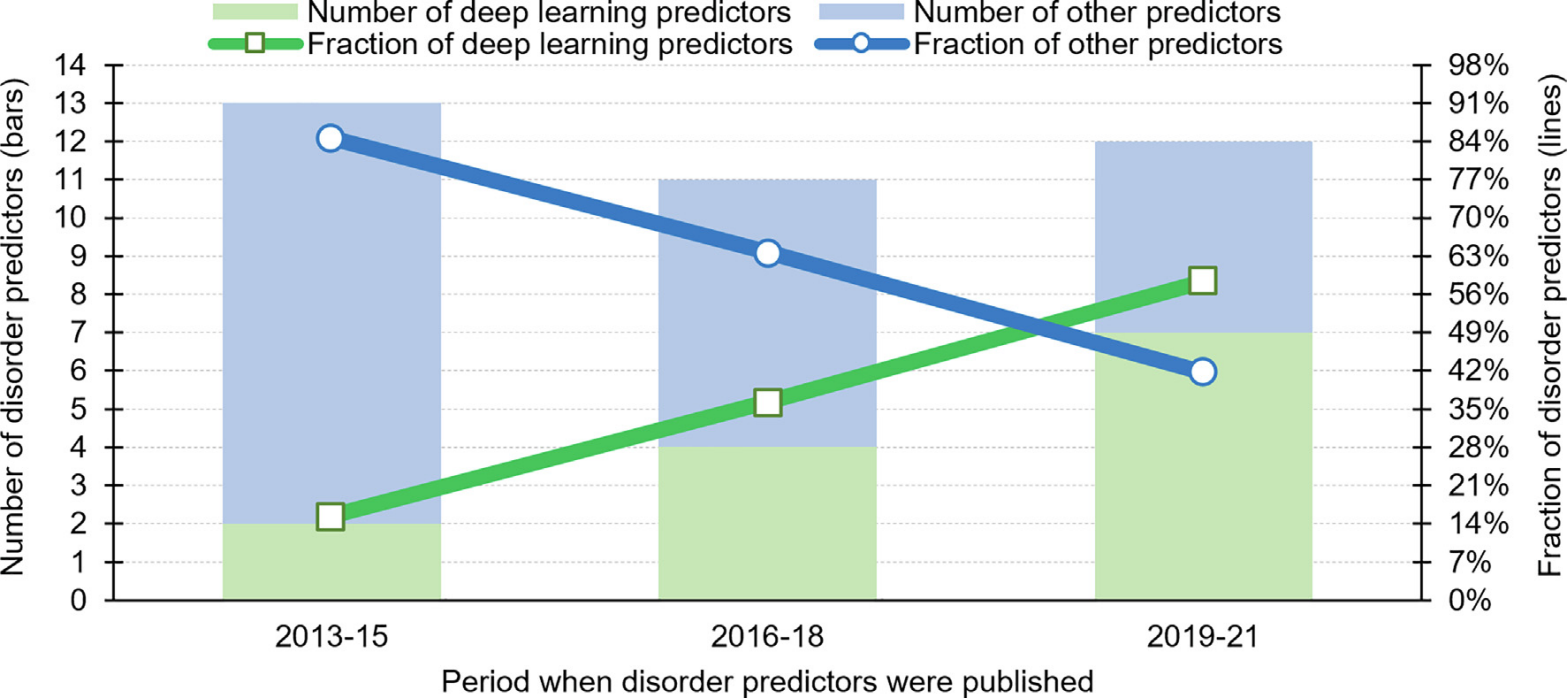
Development of disorder predictors since 2013 when the first deep learning-based predictor was released. The left/right y-axis gives the number/fraction of predictors in a given time period. The predictors are color-coded where green represents deep neural network-based methods and blue represents other types of predictors.

The intrinsic disorder prediction field was not immune to the infusion of the deep learning-based approaches. The first DNN-based disorder predictor, DNdisorder [105], was published in 2013. Table 1 summarizes a comprehensive list of 36 disorder predictors that were published since that time. This list contextualizes the efforts to develop deep learning predictors in a broader setting of the entire disorder prediction field. We identify the 36 predictors using a wide-ranging list of sources including databases of disorder predictions: MobiDB [122], D^2^P^2^ [123] and DescribePROT [124]; community assessments and surveys that were published on or after 2013 [33], [41], [42], [43], [46], [47], [49], [50], [52], [58], [59], and a manual search of relevant articles from PubMed that we collect using the “(disorder[Title]) AND (prediction[Title]) AND protein” query. Table 1 reveals that 13 out of the 36 recent disorder predictors use deep learning models. We find that it took two more years for the second DNN-based predictor, DeepCNF-D, to be published in 2015 [112]. The following three years include similarly low numbers of new deep learning tools, with two methods published in 2016, one in 2017, and one more in 2018. Year 2019 marks a turning point in the efforts to develop DNN-based disorder predictors, with two tools published in 2019, two in 2020, and four in 2021. Fig. 1 conveniently summarizes the corresponding trends. It highlights the gradual shift to developing predictors that rely on deep networks and the fact that these methods constitute majority (58%) of the predictors that were published over the last three years (green line in Fig. 1). We also note that the consistent levels of the release of new methods that range between 11 and 13 per every three-years long interval.

**Table 1:** Summary of intrinsic disorder predictors that were developed since 2013 when the first deep learning-based method was released. The predictors are sorted in the chronological order of their year of publications. “*” denotes predictors that are used in Fig. 3.

Predictor name	Year published	Reference^1^	Applies DNN	Availability^2^	URL
MFDp2	2013	[81]	No	WS	https://biomine.cs.vcu.edu/servers/MFDp2/
DNdisorder	2013	[105]	Yes	N/A	N/A
preDNdisorder	2013	[105]	No	N/A	N/A
Ulg-GIGA	2013	[106]	No	N/A	N/A
DisMeta	2014	[107]	No	WS	https://montelionelab.chem.rpi.edu/dismeta/
disCoP	2014	[77], [84]	No	WS	https://biomine.cs.vcu.edu/servers/disCoP/
DynaMine	2014	[67], [108]	No	SP + WS	https://dynamine.ibsquare.be/
PON-Diso	2014	[109]	No	WS	https://structure.bmc.lu.se/PON-Diso
DISOPRED3*	2015	[75]	No	SP + WS	https://bioinf.cs.ucl.ac.uk/psipred/
s2D-1	2015	[110]	No	No	N/A
s2D-2*	2015	[110]	No	No	N/A
DisoMCS	2015	[111]	No	N/A	N/A
DeepCNF-D	2015	[112]	Yes	SP	https://home.ttic.edu/~wangsheng/software.html
AUCpreD*	2016	[89]	Yes	N/A	N/A
AUCpreD-np*	2016	[89]	Yes	N/A	N/A
DisPredict (DisPredict2)*	2016	[66]	No	SP	https://github.com/tamjidul/DisPredict2_PSEE
MobiDB-lite*	2017	[78]	No	WS	https://mobidb.bio.unipd.it/
SPOT-Disorder*	2017	[113]	Yes	SP + WS	https://sparks-lab.org/server/spot-disorder/
IUpred2A-long*	2018	[114]	No	SP + WS	https://iupred2a.elte.hu/
IUpred2A-short*	2018	[114]	No	SP + WS	https://iupred2a.elte.hu/
pyHCA*	2018	No	No	SP	https://github.com/T-B-F/pyHCA
SPOT-Disorder-Single*	2018	[115]	Yes	SP + WS	https://sparks-lab.org/server/spot-disorder-single/
Predictor by Zhao and Xue	2018	[116]	No	No	N/A
IDP-CRF	2018	[69]	No	No	N/A
rawMSA*	2019	[88]	Yes	SP	https://bitbucket.org/clami66/rawmsa/src/master/
SPOT-Disorder2*	2019	[87]	Yes	SP + WS	https://sparks-lab.org/server/spot-disorder2/
Spark-IDPP	2019	[117]	No	No	N/A
IDP-FSP	2019	[70]	No	No	N/A
DisoMine*	2020	No	Yes	WS	https://www.bio2byte.be/b2btools/disomine/
ODiNPred	2020	[118]	No	WS	https://st-protein.chem.au.dk/odinpred
IDP-Seq2Seq*	2020	[119]	Yes	WS	https://bliulab.net/IDP-Seq2Seq/
flDPnn*	2021	[86]	Yes	SP + WS	https://biomine.cs.vcu.edu/servers/flDPnn/
flDPlr*	2021	[86]	No	No	N/A
IUPred3	2021	[63]	No	SP + WS	https://iupred3.elte.hu/
RFPR-IDP*	2021	[120]	Yes	WS	https://bliulab.net/RFPR-IDP/server
Metapredict*	2021	[121]	Yes	SP + WS	https://github.com/idptools/metapredict

1 “No” means that a given predictor was not published in a peer-reviewed journal but was included based on participation in the CASP and/or CAID assessment.

2 Availability: released as “SP” (standalone program), “WS” (web server). “No” not released as either SP (standalone program) or WS (web server), and “N/A” (not available) SP and/or WS were released at the time of publication (i.e. URL was provided in the original article) but they were not available as of February 2022 when the access was tested.

Table 1 provides a few additional insights. We manually check websites of the corresponding methods and find that 23 out of 36 predictors (over 60%) are available to the end users as either standalone software (5 methods), webserver (10 methods) or in both modalities (10 methods). Interestingly, all DNN-based predictors that were published after 2016, except for flDPlr, are among the publicly available tools. This rate of availability is substantially better compared to related areas including prediction of protein-binding and RNA-binding residues where the availability is at around 40% [103], [125]. The webservers are a convenient option to less programming savvy end users, such as some biochemists or structural biologists. In this case, predictions are performed on the webserver end and users are not required to install and run the software on their hardware. However, the main drawbacks of webservers are that they depend on the uninterrupted availability of Internet, limit the size of individual jobs (i.e., number of proteins can be predicted), and their results could be delayed when their workload is heavy. On the other hand, the standalone software option is best suited for skilled programmers and bioinformaticians. The software must be installed and executed locally. This facilitates running larger jobs and allows embedding a given disorder predictor into other bioinformatics pipelines. For instance, putative disorder generated by the popular IUPred [61], [62], [120] was used to predict DNA-binding residues [126], B-cell epitopes [127], and quality of protein structures [128].

Table 2 details the 13 deep learning-based disorder predictors. We summarize inputs, topologies, predictive performance, and runtime of these methods. The inputs cover a broad range of relevant information including the input sequence itself and several sequence-derived characteristics, such as evolutionary information (e.g., position-specific scoring matrix (PSSM) and residue-level conservation), putative structural features (e.g., secondary structure and solvent accessibility), and physiochemical characteristics that are typically quantified at the amino acid level (e.g., polarizability, hydrophobicity, and isoelectric point). We define topologies based on two key aspects: type of the deep network and its size/depth. The network types include classical deep feed forward neural networks (FFNNs) and more sophisticated restricted Boltzmann machines (RBM), convolutional neural networks (CNNs) and bidirectional recurrent neural networks (BRNNs). We grade the network sizes by the number of hidden layers into three categories: moderately deep with between 2 and 3 hidden layers; deep with 4 to 5 hidden layers; and very deep with over 5 hidden layers. We observe a few interesting patterns. First, majority of the predictors rely on multiple input types, with the two most popular options being evolutionary and putative structural data. These methods take advantages of the deep neural network’s ability to combine diverse types of inputs including numeric data, such as conservation and relative solvent accessibility, nominal data, such as secondary structure, and binary data, such as one-hot encoding of amino acid types, to produce high-quality latent feature space. Second, these disorder predictors rely on a diverse collection of network types, including hybrid designs that combine convolutional and bidirectional recurrent topologies. Third, they utilize designs with widely varying network sizes including nine moderately deep, one deep and three very deep networks. Altogether, this analysis reveals that the current designs broadly explore the input and network topology spaces.

**Table 2:** Summary of intrinsic disorder predictors that use deep neural network models. The predictors are sorted in the chronological order of their year of publications. X marks inputs that are used by a given predictor. “*” denotes predictors that are used in Fig. 3.

Predictor name	Year published	Inputs				Network architecture		AUC	Runtime^7^
		Sequence^1^	Evolutionary features^2^	Predicted structural feature^3^	Physicochemical properties^4^	Type^5^	Size^6^		
DNdisorder	2013		X	X		RBM	Moderately deep	N/A	N/A
DeepCNF-D	2015		X	X	X	CNN	Moderately deep	N/A	N/A
AUCpreD*	2016	X	X	X	X	CNN	Moderately deep	0.757	7.0
AUCpreD-np*	2016	X		X	X	CNN	Moderately deep	0.751	<0.5
SPOT-Disorder*	2017		X	X	X	BRNN	Moderately deep	0.744	5.0
SPOT-Disorder-Single*	2018	X	X		X	BRNN + CNN	Deep	0.757	0.8–1.0
rawMSA*	2019		X	X		BRNN + CNN	Very deep	0.780	>10.0
SPOT-Disorder2*	2019	X	X	X		BRNN + CNN	Very deep	0.760	>10.0
DisoMine*	2020			X		BRNN	Moderately deep	0.765	<0.5
IDP-Seq2Seq*	2020		X	X	X	BRNN	Very deep	0.754	12.0
flDPnn*	2021		X	X	X	FFNN	Moderately deep	0.814	0.5–1.0
RFPR-IDP*	2021		X		X	BRNN + CNN	Moderately deep	0.722	<0.5
Metapredict*	2021			X		BRNN	Moderately deep	0.746	<0.5

1 The input sequence was encoded and directly used as predictive input.

2 Evolutional features computed from the input sequence including position-specific scoring matrix (PSSM), entropy-based conservation, and multiple sequence alignment.

3 Structural features predicted from the input sequence, such as putative secondary structure, solvent accessibility, and half-sphere exposures.

4 Physicochemical properties of the amino acids in the input sequence including polarizability, hydrophobicity, and isoelectric point.

5 Type of the deep learning neural network used: “RBM” (Restricted Boltzmann Machine); “CNN” (Convolutional Neural Network); “BRNN” (Bidirectional Recurrent Neural Network); and “FFNN” (Feed Forward Neural Network).

6 The number of hidden layers: moderately deep with 2 to 3 layers; deep with 4 to 5 layers; and very deep with over 5 layers.

7 The average runtime in minutes to predict one amino acid sequence. N/A denotes that the results could not be collected since a working implementation of the corresponding predictor is not available.

The recently completed CAID experiment reveals that some of the DNN-based solutions provide favorable predictive performance when compared to other types of disorder predictors [52]. This conclusion is perhaps best captured with the following quote: “*The SPOT-Disorder2 and flDPnn, followed by RawMSA and AUCpreD, are consistently good. However, flDPnn is at least an order of magnitude faster than its competitors, and it succeeded on all sequences, whereas SPOT-Disorder2 skipped 5% of sequences as a result of a length limitation.*” [85]. While these four best predictors rely on deep learning, they implement the underlying predictive models using very different designs. More specifically, flDPnn relies on moderately deep FFNN architecture [86], SPOT-Disorder2 and RawMSA are very deep hybrids of CNN and BRNN [87], [88], while AUCpreD utilizes moderately deep CNN topology [89]. This observation suggests that accurate disorder prediction can be accomplished using different types of deep learners.

We provide a wider comparison of the predictive performance of deep learners. We cover 11 DNN-based methods that exclude only the two oldest methods, DNdisorder and DeepCNF-D. DNdisorder is not available to the end users (Table 1) while the standalone version of DeepCNF-D requires specific feature encoding of the sequence that we could not reproduce. We compare predictive performance of the remaining 11 deep learners using the annotated CAID dataset from https://idpcentral.org/caid/data/1/ and https://idpcentral.org/caid/data/1/reference/disprot-disorder.txt. This dataset includes 652 protein sequences and 337,908 amino acids, with 838 disordered regions and 54,820 disordered residues. For the 8 of the 11 predictors that were evaluated in CAID (i.e., AUCpred [89], AUCpred-np [89], DisoMine [129], flDPnn [86], rawMSA [88], SPOT-Disorder [113], SPOT-Disorder-Single [115] and SPOT-Disorder2 [87]), we parse their CAID predictions from https://idpcentral.org/caid/data/1/predictions/. We collect results for the other three methods (IDP-Seq2Seq [119], RFPR-IDP [120], and Metapredict [121]) using the webservers and standalone programs provided by the authors. Table 2 shows that the predictive quality of deep learners measured with the area under the ROC curve (AUC) ranges between 0.722 for RFPR-IDP and 0.814 for flDPnn.

We further evaluate whether differences in the AUCs of the 11 predictors are robust across different datasets by comparing results across 20 randomly selected disjoint sets of 5% of proteins from the CAID dataset. We assess significance of differences in AUCs between the best-performing flDPnn and the other methods. We use the *t*-test if the underlying data are normal; otherwise, we use the Wilcoxon signed-rank test; we test normality with the Anderson-Darling test at the 0.05 significance. We find that flDPnn and RawMSA are not statistically different (*p*-value ≥ 0.05) but flDPnn is statistically better than the other 9 methods (*p*-value < 0.05). We similarly quantify significance of differences between RFPR-IDP that has the lowest AUC and the other 10 predictors. This analysis reveals that SPOT-Disorder, Metapredict, AUCpreD-np and IDP-Seq2Seq produce predictions that are not statistically better than RFPR-IDP (*p*-value ≥ 0.05). The remaining 4 predictors that include AUCpreD, SPOT-Disorder-Single, SPOT-Disorder2, and DisoMine are significantly better than RFPR-IDP (*p*-value < 0.05) and significantly worse than flDPnn (*p*-value < 0.05). Correspondingly, we identify 3 groups of the DNN-based predictors: 1) flDPnn and RawMSA that secure the best results (AUC > 0.78); AUCpreD, SPOT-Disorder-Single, SPOT-Disorder2, and DisoMine that obtain the second-best performance (0.755 < AUC < 0.78); and RFPR-IDP, SPOT-Disorder, Metapredict, AUCpreD-np and IDP-Seq2Seq that provide more modest levels of predictive quality (0.720 < AUC < 0.755).

We also analyze an average per-protein runtime for the predictors from Table 2. Similar to the analysis of the predictive performance, we could not perform this analysis for DNdisorder and DeepCNF-D that do not provide working implementations. We extract the runtime data from the CAID results for the eight methods that participated in this experiment [52], and we estimate it for the other three methods (IDP-Seq2Seq, RFPR-IDP and Metapredict) based on the implementations provided by the authors. We find that the runtime of the 11 predictors varies widely (Table 2), with the fastest predictors that produce results in several seconds and the slowest that require over 10 min for the same task.

**Fig. 2:**
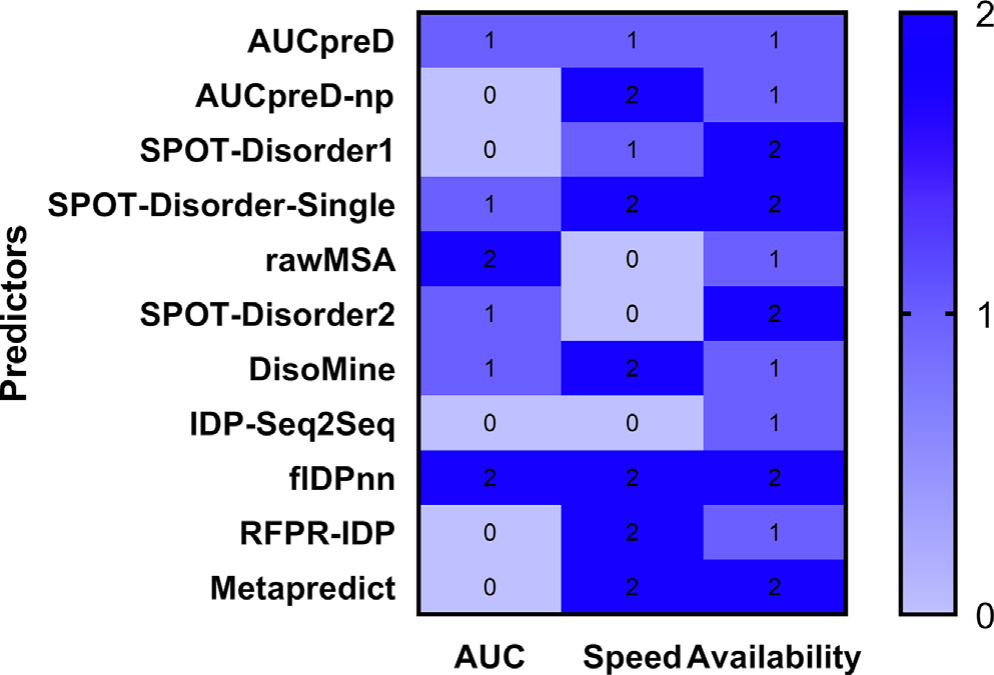
Heatmap that compares 11 available deep learners based on three key characteristics: predictive performance quantified with AUC, speed measured with runtime, and mode of availability. The predictors are sorted in the chronological order of their year of publications. The color-coded scores represent quality where 2 (dark blue) is best, 1 (blue) is intermediate, and 0 (light blue) is worst. The AUC values are categorized into three groups using statistical test that measures robustness of differences between predictors over different protein sets; details are described in the text. Methods with AUCs that are not statistically different (*p*-value ≥ 0.05) from the best (worst) performing flDPnn (RFPR-IDP) are labeled with 2 (0), while the remaining predictors are labeled with 1. The runtime is divided into three ranges: < 1 min (score of 2); between 1 and 10 min (score of 1); and ≥ 10 min (score of 0). The availability score counts the number of modes where 2 means that both SP (standalone program) or WS (web server) are available and 1 that either SP or WS are available.

Using the above analysis, Fig. 2 compares the 11 available predictors based on three key characteristics: predictive performance quantified with AUC, speed measured with runtime, and mode of availability. We score each characteristic in the 0 to 2 range where higher number is associated with darker shade and indicates better quality, i.e., higher AUC, lower runtime and more ways to access a given predictor. The most well-rounded predictors include flDPnn (total score of 6), SPOT-Disorder-Single (score of 5), DisoMine (score of 4) and Metapredict (score of 4). When analyzing individual dimensions, the fastest methods (i.e., per-protein runtime < 1 min) include AUCpreD-np, SPOT-Disorder-Single, DisoMine, flDPnn, RFPR-IDP and Metapredict. The most accurate methods are flDPnn and rawMSA and methods that are available in two modes (webserver and standalone) include SPOT-Disorder, SPOT-Disorder-Single, SPOT-Disorder2, flDPnn and Metapredict.

## 3 Deep learning methods outperform other predictors of intrinsic disorder

Motivated by the finding that the top performing predictors in CAID are deep learners [52], [85], we investigate whether this result can be extended more broadly to other DNN-based methods. More specifically, we compare the results for the 11 available deep learning-based disorder predictors from Table 2 against the results of other types of methods that we collect using the same CAID data. This analysis covers a comprehensive set of 29 disorder predictors including 11 deep learners that are annotated with * in Table 2 and 18 methods that use the other types of models. The latter group includes 12 machine learning predictors (DisEMBL-465 [36], DisEMBL-HL [36], DISOPRED3 [75], DisPredict2 [66], Espritz-D [130], Espritz-N [130], Espritz-X [130], flDPlr [86], PONDR VSL2B [131], PreDisorder [74], RONN [132], and s2D-2 [110]); 5 sequence scoring function-based methods (FoldUnfold [133], IsUnstruct [134], IUpred2A-long [114], IUpred2A-short [114], and pyHCA [135]) and one *meta*-predictor (MobiDB-lite [78]). We mark these methods with * in Table 1, except for DisEMBL-465, DisEMBL-HL, JRONN, FoldUnfold, PONDR VSL2B, PreDisorder, IsUnstruct, Espritz-D, Espritz-N, and Espritz-X that were published before 2013. We quantify the predictive performance using four popular metrics that are consistent with the measures used in the most recent community assessments [52], [58], including AUC, area under the precision-recall curve (AUPR), F1 and Matthews correlation coefficient (MCC). Finally, we quantify statistical significance of differences in the predictive performance between the results of the 11 deep learners and the 18 other methods. We test normality of the measured scores with the Anderson-Darling test and we apply the student *t*-test for normal data and the Wilcoxon test otherwise.

**Fig. 3:**
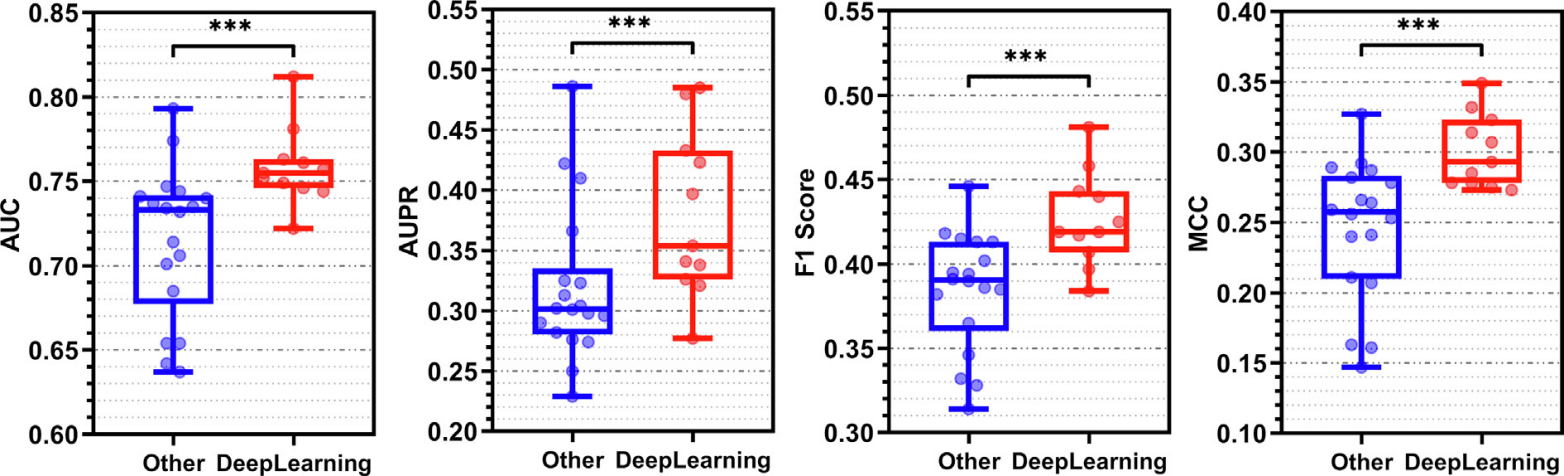
Comparison of predictive performance between disorder predictors that utilize deep neural networks (in red) and the other disorder predictors (in blue). The predictive performance is quantified with AUC, AUPR, F1 and MCC. Results of individual predictors are denoted by dots. Distributions of these values are summarized with the box plots. *** means that the predictive performance of the deep learners is significantly higher than the performance of the other methods (*p*-value < 0.05).

Fig. 3 summarizes the corresponding empirical results. The median AUC of the deep learners is 0.76 vs. 0.73 for the other tools. We observe similarly substantial magnitude of differences for the other metrics, with median AUPR of 0.35 vs. 0.31, median F1 of 0.42 vs. 0.39 and median MCC of 0.29 vs. 0.26. The statistical analysis reveals that the DNN-based methods outperform the other disorder predictors by a statistically significant margin across the four metrics (*p*-value < 0.05). This consistent and statistically significant trend suggests that the deep neural networks are the best choice to develop accurate disorder predictors.

## 4 Summary and outlook

Disorder prediction is an active and well-establish research area with over 40 years of history. The first DNN-based disorder predictor was published in 2013 and 12 more deep learners were published since. We find that majority of the disorder predictors that were developed in the last three years utilize deep neural networks. The popularity of this design is motivated by several factors. First, these models can be molded into many different architectures that are flexible to use diverse types of inputs. Our analysis of the 13 DNN-based disorder predictors reveals that they rely on very diverse designs that explore different inputs, topologies and sizes. Second, our empirical results reveal that the DNN-based predictors are in general statistically better when directly compared against a representative collection of the other types of predictive models. This conclusion is in line with the results of the recent CAID experiment where the top four predictors are deep learners [52], [85]. Third, our multifaceted comparison of the deep learners provides useful clues for the end users by identifying methods that are accurate, fast and widely available. We identify several well-rounded predictors that include flDPnn (very accurate, very fast, and available in multiple ways), SPOT-Disorder-Single (accurate, very fast, and available in multiple ways), DisoMine (accurate and very fast) and Metapredict (very fast and available in multiple ways). These results and accolades support conclusions of the a recent article that say “*deep-learning-based methods will likely continue to show the greatest potential for future improvement*” [85].

Our analysis finds that the architectures of the current deep learners are considerably diverse. This suggests that the optimal architecture is yet to be identified. We reason that this should be a hybrid design to accommodate for the underlying variety of different types/flavors of disorder [136], [137], [138]. For instance, IDRs cover a wide spectrum of sizes, from short regions that are frequently localized at the sequence termini to very long regions that span the entire protein sequence [139], [140]. IDRs also vary in their conformational space, which is signified by their classification into the native coils, native pre-molten globules and native molten globules [4], [141]. Moreover, IDRs carry out many different functions, and some of them are multifunctional (moonlighting) [142], [143], which results in many different biases in their sequences [4], [137]. Interestingly, design of the recently published and well-rounded flDPnn suggests that predictive quality can be improved by innovating inputs that are fed into the deep networks [86]. The authors point to multiple options including development of extended sequences profiles that cover relevant sequence-derived protein characteristics beyond the commonly-used inputs listed in Table 2, and construction of aggregate features that quantify sequence bias at the region or whole sequence level. These two future directions go hand in hand given the fact that the hybrid deep learners are inherently capable of handling diverse and large inputs.

While most of recently released predictors of intrinsic disorder utilize DNNs, this is not necessarily the case for the methods that predict binding IDRs. There are close to 20 predictors of disordered protein-binding regions [144] and several methods that predict IDRs that interact with nucleic acids and lipids [42], [145]. Examples of the recently published tools include FLIPPER [146], SPOT-MoRF [147], OPAL+ [148], DisoLipPred [149] and DeepDISObind [150]. The CAID experiment evaluated close to a dozen of these predictors and concluded that “*disordered binding regions remain hard to predict*” [52], motivating further efforts in this area. One of the potential reasons for the low predictive performance of these tools is a relatively low utilization of the deep learning architectures. We identify only a handful of DNN-based predictors of binding IDRs including SPOT-MoRF [147], MoRFPred_en [151], en_DCNNMoRF [152], DeepDISObind [150], and DisoLipPred [149]. A similar situation is true in the context of prediction of disordered linker regions where neither of the two currently available methods, DFLpred [153] and APOD [154], applies deep learning and their predictive performance is relatively limited. Given the success of DNNs in the disorder prediction, we believe that this technology could be successfully applied to strengthen the quality of the predictors of binding IDRs and disordered linkers.

## Funding

This research was funded in part by the National Science Foundation (grant 2125218) and the Robert J. Mattauch Endowment funds to L.K.

## CRediT authorship contribution statement

**Bi Zhao:** Formal analysis, Data curation, Investigation, Validation, Writing – original draft, Writing – review & editing. **Lukasz Kurgan:** Conceptualization, Formal analysis, Funding acquisition, Project administration, Supervision, Validation, Writing – original draft, Writing – review & editing.

## Declaration of Competing Interest

The authors declare that they have no known competing financial interests or personal relationships that could have appeared to influence the work reported in this paper.
